# Supplementary material for: Memory-guided microsaccades
Source: Nat Commun. 2019 Aug 16;10:3710. doi: 10.1038/s41467-019-11711-x (PMC6697692; doi:10.1038/s41467-019-11711-x)
Supplement: Supplementary file 1 — Supplementary Information [file 41467_2019_11711_MOESM1_ESM.pdf]

Memory-guided microsaccades

Willeke*, Tian*, Buonocore* et al.

Memory-guided microsaccades

Konstantin F. Willeke*, Xiaoguang Tian*, Antimo Buonocore*, Joachim Bellet, Araceli Ramirez-Cardenas, and Ziad M. Hafed

Supplementary information

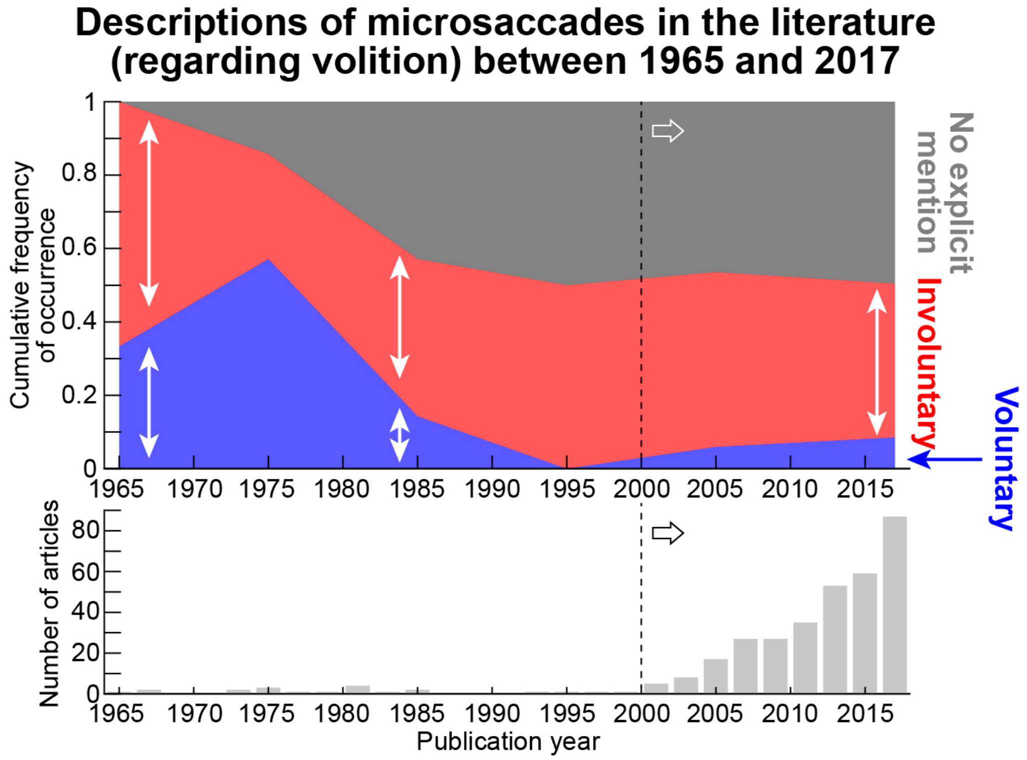

Supplementary Figure 1 Overwhelming description of microsaccades as being involuntary eye movements in the literature. In the top panel, we performed a meta-analysis of all published articles on microsaccades between 1965 and 2017. We classified articles based on how they referred to microsaccades with respect to volition (see Methods). We grouped the articles into 3 categories (Methods): explicitly referring to microsaccades as being either voluntary (blue) or involuntary (red) eye movements, or as making no explicit mention regarding volition (dark gray). Each time slice (x-axis) shows the distribution of these 3 categories in published research (y-axis). The bottom histogram shows the number of published articles in 2-year bins within the same time period as in the top panel; it shows a pattern consistent with Rolfs' meta-analysis[1], in which there was almost a complete gap of research on microsaccades in the final two decades of the previous century. In practically all time slices of the top panel, there was a majority of references to microsaccades as being involuntary compared to voluntary. In the current century (dashed vertical line and rightward block arrows), there was a significant increase in microsaccade research (bottom histogram) with a concomitant introduction of a large bias to refer to microsaccades as being involuntary. The large fraction of gray papers (with no explicit mention regarding volition) in the current century is attributed to the emergence of new questions regarding microsaccades[2], such as their links with cognition.

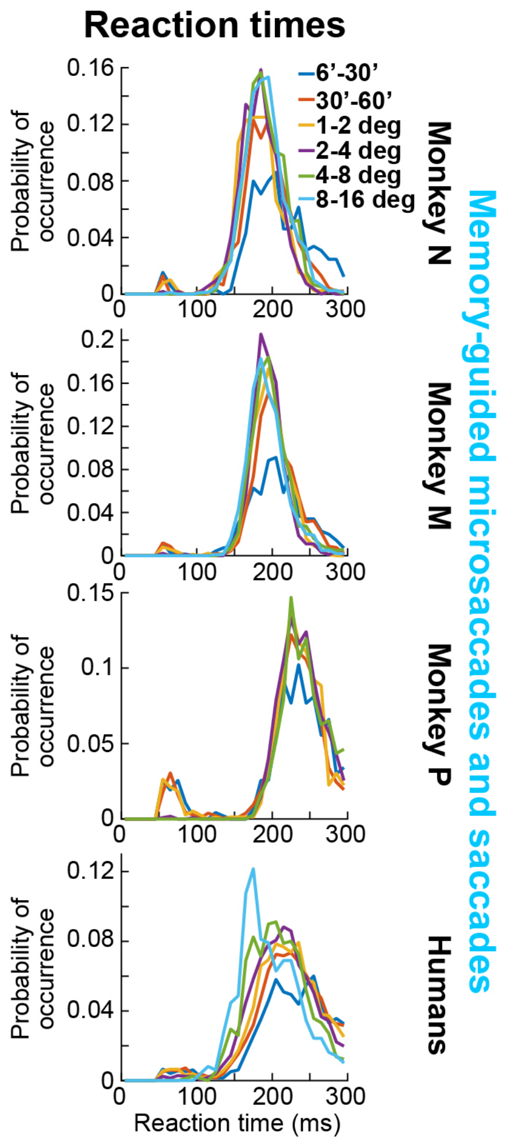

Supplementary Figure 2 Similarity of memory-guided microsaccade reaction times to the reaction times of larger memory-guided saccades. In each panel, we show data similar in format to Fig. 2a, d, g, j. Here, we included large saccades, and we also binned data according to memory-guided saccade/microsaccade size (see color legend in the top panel). Memory-guided microsaccadic reaction times (the first two colors in the color legend; movements with <60 min arc amplitudes) had similar distributions to those of larger memory-guided saccades. Note that there were quantitative differences in reaction times (particularly evident in the human subjects in which we had larger numbers of trials overall) that reflect factors other than the intrinsic ability to react to the instruction to generate an eye movement (for example, increases in reaction times for smaller target eccentricities[3]).

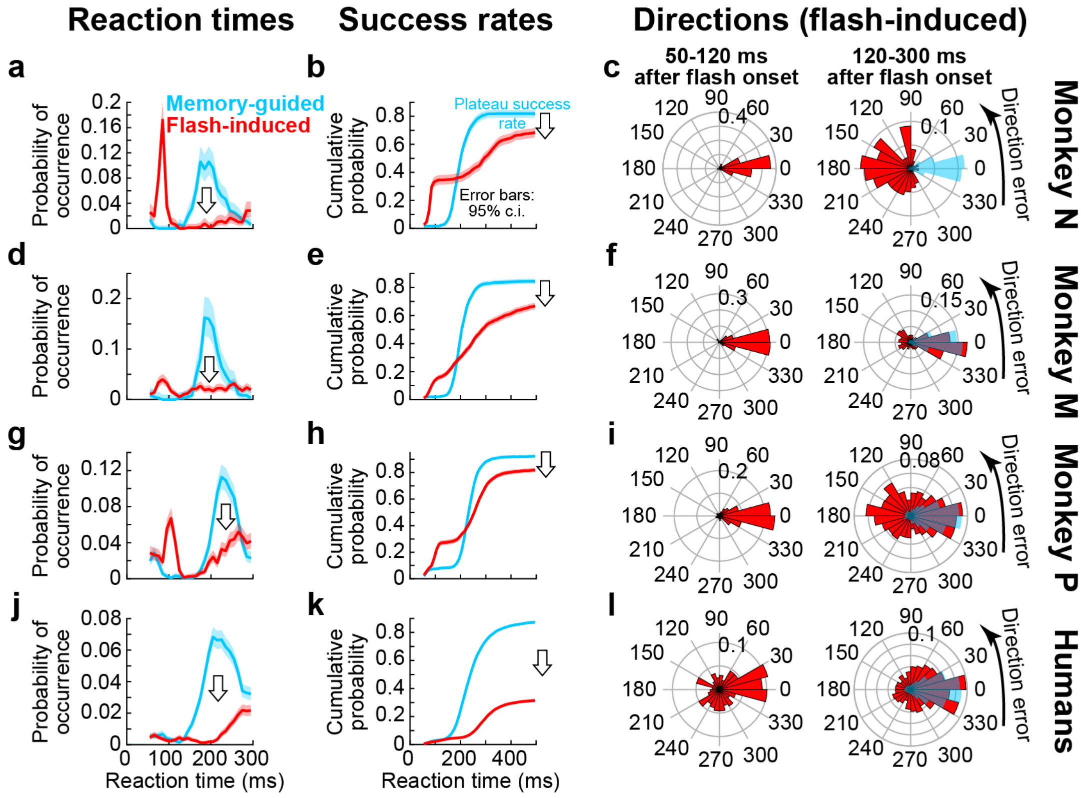

Supplementary Figure 3 Memory-guided microsaccades were more successful and directionally accurate than flash-induced microsaccades at the beginnings of trials. For the same data as in Figs. 2, 3, we plotted reaction time (**a**, **d**, **g**, **j**), success rate (**b**, **e**, **h**, **k**), and direction error (**c**, **f**, **i**, **l**) for the first microsaccade to occur after target flash onset (flash-induced microsaccades; red). The monkeys exhibited express reactions[4] on a minority of trials (**a**, **d**, **g**, **j**), similar to the corrective, visually-guided movements in Fig. 2m, p, s, but then barely any eye movement responses at all at the times in which the instructed memory-guided microsaccades would have happened (the light blue histograms show the memory-guided microsaccade reaction time distributions for easy comparison). Humans had no express reactions under the presented conditions, but they again showed barely any responses to target flash onset in the later interval in which memory-guided microsaccades were triggered (also see Fig. 1). This means that the overall success rates after target flash onset were significantly lower than for the instructed memory-guided microsaccades (**b**, **e**, **h**, **k**). Therefore, the latter movements did not reflect transient reflexes, perhaps due to covert attentional shifts[5,6] associated with the task. In terms of direction errors, we plotted angular distributions (as in Fig. 3) for flash-induced microsaccades (**c**, **f**, **i**, **l**). In the left column of each panel, we plotted direction errors for express reactions (occurring 50-120 ms after target flash onset), and in the right column of each panel, we plotted the direction errors for reaction times 120-300 ms after target flash onset (similar to the range of reaction times for instructed memory-guided microsaccades). Express flash-induced microsaccades were directionally accurate, as expected[4]; the later responses were significantly less directionally accurate than instructed memory-guided microsaccades (shown in transparent light blue for easier comparison). Error bars denote 95% c. i.

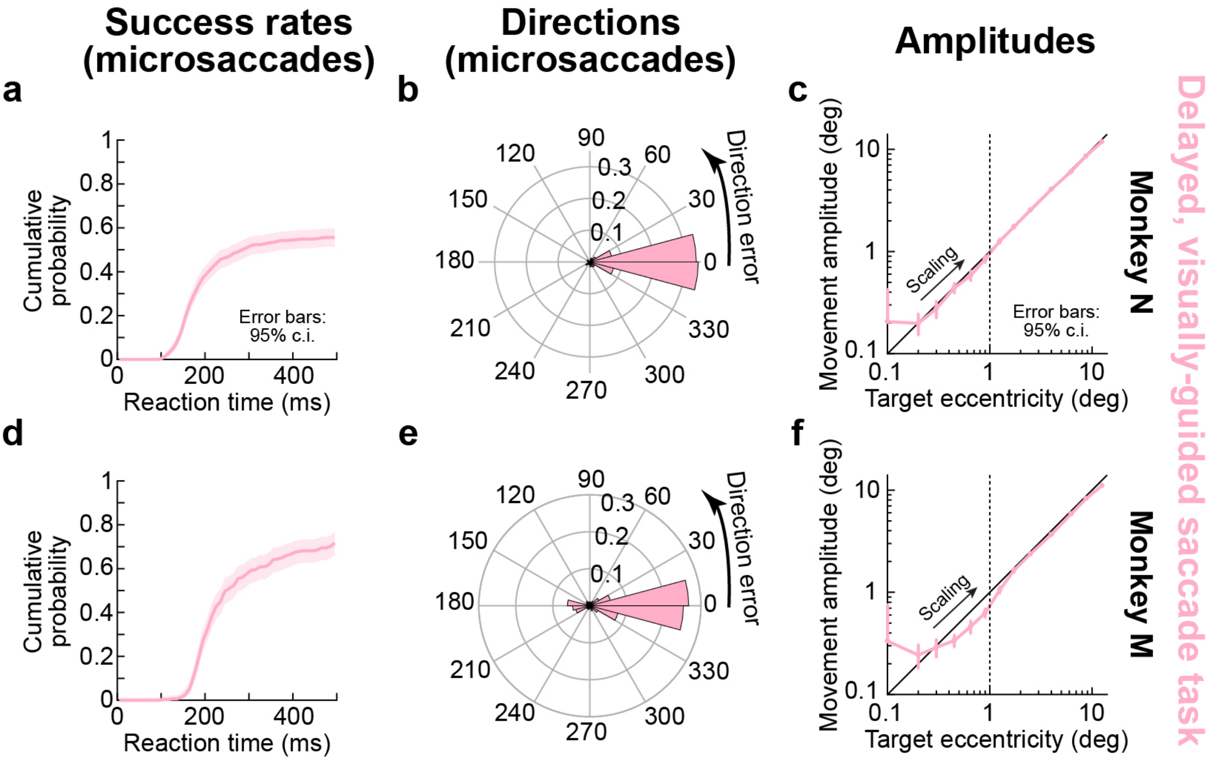

Supplementary Figure 4 Delayed, visually-guided microsaccades had lower success rates than memory-guided microsaccades, but they were spatially accurate in both direction and amplitude, as expected. (a) Success rate as in Fig. 2b for monkey N in the delayed, visually-guided saccade task. The shown data is for all trials with target eccentricities <1°. The monkey often failed to generate the instructed movement within the specified time, resulting in a plateau success rate of only ~50% (significantly less than for instructed memory-guided microsaccades). This could reflect an overall difficulty in withholding eye movement responses for prolonged periods with the continuous presence of a visual target; the monkey often just fixated the persistently present visual target before the go instruction. **(b)** However, the successful microsaccades that were generated in the task were directionally accurate. We plotted the distribution of angular direction errors between target locations and evoked eye movements (as in Fig. 3). A distribution centered near 0 direction error indicates directionally accurate eye movements. **(c)** The successful microsaccades were also accurate in terms of their amplitude relative to target eccentricity. No overshoot was observed as in the case of memory-guided microsaccades and uninstructed corrective, visually-guided microsaccades (Fig. 4) (and also memory-guided perceptual judgements; Fig. 5). **(d, e, f)** Similar observations for monkey M. n=581 trials for **a**, **b**; n=2843 trials for **c**; n=314 trials for **d**, **e**; n=2476 trials for **f**. Error bars denote 95% c. i.

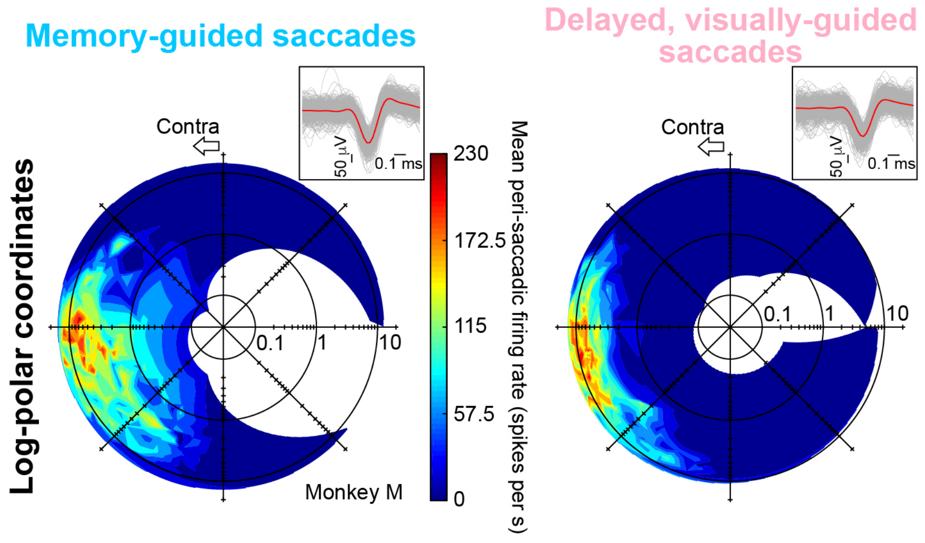

Supplementary Figure 5 The activity of the neuron of Fig. 7a, b in different tasks. The left panel shows the neuron's activity from the memory-guided saccade task, including for large saccades, and the right panel shows the neuron's activity from the delayed, visually-guided saccade task (and with a similar range of tested saccade directions and amplitudes). All formatting conventions are similar to Figs. 6, 7. During the memory-guided saccade task (left), the neuron's movement RF expanded foveally, allowing the neuron to respond exclusively for memory-guided microsaccades but not for similarly sized visually-guided microsaccades (Fig. 7a, b). The insets show individual spike waveforms from the neuron for every tenth spike in each task (light gray), with the red waveforms being the averages (of ~700 spikes each). We ensured that the neuron met criteria for being sorted as the same neuron in the two tasks (e.g. inter-spike intervals and waveform shape criteria; Methods). Each inset shows ~70 waveforms (gray curves) per task.

1 Rolfs, M. Microsaccades: small steps on a long way. *Vision Res* **49**, 2415-2441 (2009).

2 Hafed, Z. M. Mechanisms for generating and compensating for the smallest possible saccades. *Eur J Neurosci* **33**, 2101-2113 (2011).

3 Kalesnykas, R. P. & Hallett, P. E. Retinal eccentricity and the latency of eye saccades. *Vision Res* **34**, 517-531 (1994).

4 Tian, X., Yoshida, M. & Hafed, Z. M. Dynamics of fixational eye position and microsaccades during spatial cueing: the case of express microsaccades. *J Neurophysiol* **119**, 1962-1980 (2018).

5 Hafed, Z. M. & Clark, J. J. Microsaccades as an overt measure of covert attention shifts. *Vision Res* **42**, 2533-2545 (2002).

6 Engbert, R. & Kliegl, R. Microsaccades uncover the orientation of covert attention. *Vision Res* **43**, 1035-1045 (2003).
