# Supplementary material for: Memory-guided microsaccades
Source: Nat Commun. 2019 Aug 16;10:3710. doi: 10.1038/s41467-019-11711-x (PMC6697692; doi:10.1038/s41467-019-11711-x)
Supplement: Supplementary file 3 — Reporting Summary [file 41467_2019_11711_MOESM3_ESM.pdf]

nature research

Corresponding author(s): Ziad M. Hafed

Last updated by author(s): Jul 16, 2019

Reporting Summary

Nature Research wishes to improve the reproducibility of the work that we publish. This form provides structure for consistency and transparency in reporting. For further information on Nature Research policies, see Authors & Referees and the Editorial Policy Checklist.

Statistics

For all statistical analyses, confirm that the following items are present in the figure legend, table legend, main text, or Methods section.

n/a	Confirmed	
☐	☒	The exact sample size (*n*) for each experimental group/condition, given as a discrete number and unit of measurement
☐	☒	A statement on whether measurements were taken from distinct samples or whether the same sample was measured repeatedly
☐	☒	The statistical test(s) used AND whether they are one- or two-sided<br>*Only common tests should be described solely by name; describe more complex techniques in the Methods section.*
☒	☐	A description of all covariates tested
☐	☒	A description of any assumptions or corrections, such as tests of normality and adjustment for multiple comparisons
☐	☒	A full description of the statistical parameters including central tendency (e.g. means) or other basic estimates (e.g. regression coefficient) AND variation (e.g. standard deviation) or associated estimates of uncertainty (e.g. confidence intervals)
☐	☒	For null hypothesis testing, the test statistic (e.g. F, t, r) with confidence intervals, effect sizes, degrees of freedom and P value noted<br>*Give P values as exact values whenever suitable.*
☒	☐	For Bayesian analysis, information on the choice of priors and Markov chain Monte Carlo settings
☒	☐	For hierarchical and complex designs, identification of the appropriate level for tests and full reporting of outcomes
☒	☐	Estimates of effect sizes (e.g. Cohen's *d*, Pearson's *r*), indicating how they were calculated

Our web collection on statistics for biologists contains articles on many of the points above.

Software and code

Policy information about availability of computer code

Data collection	EyeLink 1000, SR Research; Multichannel acquisition processor (MAP, Plexon, Inc.)
Data analysis	Matlab version 2016b, Matlab version 2018a (The Mathworks)

For manuscripts utilizing custom algorithms or software that are central to the research but not yet described in published literature, software must be made available to editors/reviewers. We strongly encourage code deposition in a community repository (e.g. GitHub). See the Nature Research guidelines for submitting code & software for further information.

Data

Policy information about availability of data

All manuscripts must include a data availability statement. This statement should provide the following information, where applicable:

- Accession codes, unique identifiers, or web links for publicly available datasets
- A list of figures that have associated raw data
- A description of any restrictions on data availability

All data presented in this paper are stored in institute computers and are available upon reasonable request.

Field-specific reporting

Please select the one below that is the best fit for your research. If you are not sure, read the appropriate sections before making your selection.

☒ Life sciences ☐ Behavioural & social sciences ☐ Ecological, evolutionary & environmental sciences

For a reference copy of the document with all sections, see nature.com/documents/nr-reporting-summary-flat.pdf

Life sciences study design

All studies must disclose on these points even when the disclosure is negative.

Sample size	We collected neurophysiological data from 2 macaque monkeys, and behavioral data from 3 macaque monkeys. 3 monkeys is higher than the minimum established standard of 2 (converged upon by the field as a compromise given the 3R principle). A total of 13 human volunteers participated in the human psychophysics experiments. In each psychophysics experiment, 7 human subjects participated; 3 were authors, 4 were naive participants. 4 of the participants completed all psychophysics experiments. The number of seven per experiment was not determined based on a numerical analysis but chosen based on previous publications with similar experimental design as well as allowing for enough repetitions of each condition for statistical analyses. Since our results are so consistent across humans and monkeys, the number is deemed sufficient.
Data exclusions	We established exclusion criteria prior to the analysis. No matter the eye-tracking recording technique, we ensured that all key-trial epochs were free of recording artifacts, blinks, and large or small saccades. We removed all trials that met these criteria. All details are provided in the paper.
Replication	We explicitly tried to replicate the finding of the memory-guided saccade task by repeating the same task with a different response modality (i.e. the 'mouse' and the 'button-task'). We also replicated findings across individual monkeys and human subjects.
Randomization	In all of the experiments, the trial order was fully randomized, such that saccade targets with larger and small eccentricities were interleaved. Targets were selected from predetermined locations that spanned 6 min arc to 12-15°.
Blinding	There was no experimental manipulation across groups. The same verbal instruction was given to each participant (and same training routine was applied to monkeys), making blinding superfluous.

Reporting for specific materials, systems and methods

We require information from authors about some types of materials, experimental systems and methods used in many studies. Here, indicate whether each material, system or method listed is relevant to your study. If you are not sure if a list item applies to your research, read the appropriate section before selecting a response.

Materials & experimental systems

n/a	Involved in the study	
☒	☐	Antibodies
☒	☐	Eukaryotic cell lines
☒	☐	Palaeontology
☐	☒	Animals and other organisms
☐	☒	Human research participants
☒	☐	Clinical data

Methods

n/a	Involved in the study	
☒	☐	ChIP-seq
☒	☐	Flow cytometry
☒	☐	MRI-based neuroimaging

Animals and other organisms

Policy information about studies involving animals; ARRIVE guidelines recommended for reporting animal research

Laboratory animals	3 male macaque monkeys (macaca mulatta), 10, 10, and 7 years of age at the time of data recording.
Wild animals	The study did not involve wild animals.
Field-collected samples	The study did not involve samples collected from the field.
Ethics oversight	All monkey experiments were approved by ethics comittees at the Regierungspräsidium Tübingen.

Note that full information on the approval of the study protocol must also be provided in the manuscript.

Human research participants

Policy information about studies involving human research participants

Population characteristics	See 'Sample Size' above.
Recruitment	Subjects were recruited from the University of Tübingen through personal communication.
Ethics oversight	Experiments were approved by ethics committees at the Medical Faculty of Tuebingen University.

Note that full information on the approval of the study protocol must also be provided in the manuscript.
