# Supplementary material for: Memory-guided microsaccades
Source: Nat Commun. 2019 Aug 16;10:3710. doi: 10.1038/s41467-019-11711-x (PMC6697692; doi:10.1038/s41467-019-11711-x)
Supplement: Supplementary file 4 — Description of Additional Supplementary Files [file 41467_2019_11711_MOESM4_ESM.pdf]

Description of Additional Supplementary Files

File Name: Supplementary Movie 1
Description: **Example trial showing the instantaneous eye position of a human subject generating a voluntary, memory-guided microsaccade towards an invisible foveal location.** The cyan cross depicts instantaneous eye position, and the green dashed circle around the central fixation point (white dot) is a circle of 1o radius. The scale bar on the bottom left describes visual dimensions of the displayed area, and the bottom right text indicates elapsed time from trial onset. The movie is running in slow motion to clarify the specific task events, which are highlighted by the text on the top left corner of each frame. The movie pauses briefly at target flash onset (normally a very brief event) in order to demonstrate the small eccentricity associated with the memory-guided microsaccade that is successfully generated later. The subject generated a spatially accurate microsaccade to the invisible target location after the fixation spot disappearance (the go command). Supplementary Movie 2 shows the same movie in real-time.

File Name: Supplementary Movie 2
Description: **Same as Supplementary Movie 1, but now running in real-time.** Despite the rapid sequence of events in the trial, the subject was successfully able to voluntarily generate a spatially accurate memory-guided microsaccade.
